# A scoping review on quality assessment tools used in systematic reviews and meta-analysis of real-world studies

**DOI:** 10.1007/s00296-023-05354-x

**Published:** 2023-06-16

**Authors:** Tadesse Gebrye, Francis Fatoye, Chidozie Mbada, Zalmai Hakimi

**Affiliations:** 1 Department of Health Professions, Faculty of Health, Psychology, and Social Care, Manchester Metropolitan University, Brooks Building, Birley Fields Campus, Bonsall Street, 53 Bonsall Street, Manchester, M15 6GX UK; 2 Lifestyle Diseases, Faculty of Health Sciences, North-West University, Mahikeng, South Africa; 3 Apellis Pharmaceuticals, Zug, Switzerland

**Keywords:** Quality assessment tool, Real-world data, Systematic review, Meta-analysis

## Abstract

Risk of bias tools is important in identifying inherent methodical flaws and for generating evidence in studies involving systematic reviews (SRs) and meta-analyses (MAs), hence the need for sensitive and study-specific tools. This study aimed to review quality assessment (QA) tools used in SRs and MAs involving real-world data. Electronic databases involving PubMed, Allied and Complementary Medicine Database, Cumulated Index to Nursing and Allied Health Literature, and MEDLINE were searched for SRs and MAs involving real-world data. Search was delimited to articles published in English, and between inception to 20 of November 2022 following the SRs and MAs extension for scoping checklist. Sixteen articles on real-world data published between 2016 and 2021 that reported their methodological quality met the inclusion criteria. Seven of these articles were observational studies, while the others were of interventional type. Overall, 16 QA tools were identified. Except one, all the QA tools employed in SRs and MAs involving real-world data are generic, and only three of these were validated. Generic QA tools are mostly used for real-world data SRs and MAs, while no validated and reliable specific tool currently exist. Thus, there is need for a standardized and specific QA tool of SRs and MAs for real-world data.

**Supplementary Information:**

The online version contains supplementary material available at 10.1007/s00296-023-05354-x.

## Introduction

Systematic Reviews (SRs), evidence-based medicine, and clinical guidelines bring together trustworthy information by systematically acquiring, analysing, and transferring research findings into clinical, management, and policy arenas [1]. As such, findings of different work in medical literature on related topics are evaluated using SRs and meta-analyses (MAs), through the application of scientific strategies that limit bias and errors that occur by chance [2]. Availability of the best evidence obtained though SRs and MAs is necessary to help clinicians, policy makers and patients reach the best health care decisions [3]. However, SRs and MAs require resources, take time, and are labour-intensive, as well, they may not always be warranted or possible. For example, a study estimated the expense of SRs for academic institutions and pharmaceutical companies to cost approximately $141,194.80, and on average, the total cost of all SRs per year to academic institutions and pharmaceutical companies amounts to $18,660,304.77 and $16,761,234.71 [4]. Therefore, unnecessary duplication of SRs should be avoided for cost, as well as given the large unmet need for SRs of a wide range of questions and the need to keep reviews up-to-date [5].

To use the results of SRs and MAs, it is important to assess the methodological quality of the primary studies [6]. Methodological quality assessment (QA) is the process of assessing the design and conduct of the included studies, and it is useful to establish transparency of evidence synthesis and to guarantee the certainty of the body of evidence of the review objective [7, 8]. The main reason for assessing methodological quality of primary studies is to identify risks of bias [9] which may be due to poor reporting and several design features that are dependent on the research question. Poor reporting may prevent assessment of key features of design, making it difficult to evaluate whether the study methodology has been adequate [10]. According to National Health and Medical Research Council [11], “risks of bias refer to the likelihood that features of the study design or conduct of the study will give misleading results”, and thus bring about misused resources, un-thriftiness for effective interventions or harm to consumers [11].

A systematic review of methodological assessment tools for preclinical and clinical studies, and clinical practice guidelines show that there are a variety of methodological assessment tools for different types of study design [12]. Thus, it is critical to identify the study type before choosing the corresponding QA tool. In accordance, Zeng and colleagues [12] submit that further efforts in the development of critical appraisal tools are warranted for areas that currently lack such tools. However, there is an apparent dearth of specific QA tool for real-world evidence (RWE) studies. According to Food and Drugs Administrations [13], “RWE is the clinical evidence about the usage and potential benefits, or risks of a medical product derived from analysis of real-world data (RWD)”. Whereas RWD are routinely collected data pertaining to health status and/or health care delivery of the patient which are collected from a range of sources” [14] including claims, clinical studies, clinical setting, pharmaceuticals, and patient-powered platforms [15, 16].

The increasing use of electronic health records, and health information systems has led to repositories of large volumes of complex longitudinal RWD [17]. Thus, RWD are mostly diversified, but generally are medical records, prescription data and lifestyle-related information from health care providers, hospitals, and pharmacies [18]. For primary studies based on RWD, the quality of their data should be defined in context, clearly represented, and accessible [15, 19]. For example, Hyrich [20] concludes that RWD plays significant role in rheumatology because it helps to better understand disease progression and treatment outcomes beyond the conclusions of a clinical trial, as it provides a platform to "test" outcomes in an uncontrolled, real-life environment. Furthermore, the author posits that there is need to generate trustworthy conclusions from RWD by ensuring appropriate methodological and ethical considerations for handling RWD. Given the importance of RWD in research, population health, quality improvement, clinical decision support, and personalised medicine [21], it is necessary to explore the existing QA tools that have been used for SRs and MAs that involved RWD. Hence, this scoping review of QA tools used for SRs and MAs that involved RWD.

## Methods

### Scoping review

We conducted a scoping review, a type of literature review that is used when it is difficult to identify a narrow review question; no prior synthesis has been undertaken on the topic; studies in the review sources are likely to have employed a range of data collection and analysis techniques; and a quality assessment of reviewed sources is not going to be conducted [22].

### Search strategy

An electronic database search was carried out by the reviewers through November 2022 using the following databases: PubMed, Allied and Complementary Medicine Database (AMED), Cumulated Index to Nursing and Allied Health Literature (CINAHL), and MEDLINE. The keywords used in the search included a combination of RWE, RWD, routinely collected data, electronic health records, claims and billing activities, registries, meta‐analysis, and systematic review (Appendix 2). Further, a manual search of reference sections of the included studies was also checked for additional studies. The search was delimited to articles published in English language.

### Study selection and data extraction

One reviewer screened the abstracts of all publications obtained by the search strategies. Studies meeting the following inclusion criteria were selected for further review: interventional or observational studies, using real-world data, employed methodological QA tools. SRs or MAs not based on RWD and not methodological quality assessed were excluded. The potential eligible papers were retrieved, and the full articles were obtained and assessed for their relevance by two reviewers (TG & CEM) based on the preplanned criteria for inclusion. Any disagreement in study selection was resolved through discussion and consultation with a third reviewer (FF) where necessary.

A summary table was used to display the extracted data. The following data were extracted: authors and date, type of study, type of QA tool, number of items, domains, whether the tool is generic or specific, time to complete the tool, psychometric properties (validity and reliability), population/studies used to validate the tool, and name of the unit that developed the tool. The reviewers resolved differences through discussion to achieve consensus.

### Data synthesis

Study data were extracted by three reviewers into a template. Findings for each study focusing on the QA tools used in SRs and MAs of RWD were then summarized by one reviewer, and the summaries discussed and modified by the research team as necessary, to generate an overall conclusion about the quality assessment (QA) tools used in SRs and MAs involving real-world data.

## Results

**Fig. 1:**
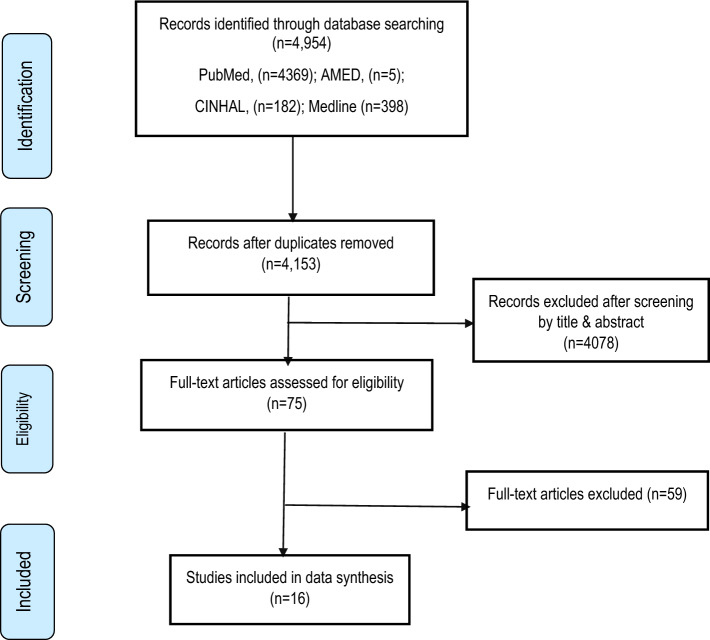
Flow diagram of publications included and excluded in the review

The search strategy retrieved 4,954 (PubMed = 4369; AMED = 5; CINHAL = 182; Medline = 398) articles from four databases (Fig. 1). After duplicates removal, the tittles, and abstracts of 4,153 publications were screened. From this, only 75 studies were included for full-text screening and 16 articles met the inclusion criteria.

### Characteristics of included studies

The characteristics of the included studies are presented in Table 1. The included studies were published between 2016 and December 2021. Seven of the included studies were observational type and the remaining were interventional and observational type of studies. The included studies applied various QA tools. The number of items used for QA within the included studies ranged from 4 to 22. Seven of the included studies comprised core domains that contains different questions employed for quality assessment. Only one [23] of the included studies utilised very specific tools for methodological quality assessment. Three [24–26] of the included studies employed validated QA tools. In order to validate the tools used in the included studies, they employed 39 non-randomised studies [24], 131 cohort studies [25] and 30 cost-effectiveness studies [26]. On the other hand, the QA tools utilised to the remaining thirteen of the included studies were not validated.

**Table 1:** Characteristics of the tools used in the included studies

Authors (years)			Type of study		Type of tool				Number of items		Scale		Domains				Generic/specific			Validity/reliability			Population/studies used for validation		Developmental unit		
Halling et al. [27]			Intervention and observational studies		QA Tool for observational cohort and cross-sectional Studies				14		Good, fair, or poor		N/A				Generic			Not validated			The tool is still developing		National Institutes of Health; Bethesda, USA^Ω^		
Coratti et al. [24]			Intervention and observational studies		A risk of bias assessment tool for non-randomized studies				6		Low, high, or unclear		N/A				Generic			Validated			39 nonrandomised studies		National Evidence-Based Healthcare Collaborating Agency and the Health Insurance Review and Assessment Service of the Re-public of Korea		
Hidayat et al. [28]			Intervention and observational studies		The Newcastle Ottawa Scale (NOS): nonrandomised studies				9		Low, moderate or high		Yes (*n* = 3)				Generic			Validated			131 cohort studies included in eight meta-analyses		Ongoing collaboration between the Universities of Newcastle, Australia and Ottawa, Canada		
Kolmos et al. [29]			Observational studies		CASP checklist for cohort studies, prospective—or retrospective registry studies				12		Yes, can't tell, no		Yes (*n* = 11)				Generic			Not validated			NA		CASP checklists were developed in 1993		
Alipour et al. [25]			Observational studies		The Newcastle Ottawa Scale (NOS): randomised controlled trials and studies reporting non-randomised controlled comparisons				9		Low, moderate, or high quality		Yes (*n* = 3)				Generic			Validated			131 cohort studies included in eight meta-analyses		Ongoing collaboration between the Universities of Newcastle, Australia and Ottawa, Canada		
Evans et al. [23]		Observational studies				Non-summative four-point system for large databases and registries		4		Adequate, not adequate or not reported				N/A				Specific	Not validated			N/A				Wylde and colleagues	
van der List et al. [30]		Intervention and observational studies				GRADE: retrospective and prospective cohort studies		12		Low risk, some concerns, high risk				N/A				Generic	Not validated			N/A				The GRADE Working Group	
Rahhal et al. [31]		Intervention and observational studies				ROBINS-I: observational non-randomized studies		7		Low, moderate, serious, critical risk of bias and no information				N/A				Generic	Not validated			N/A				Members of the Cochrane Bias Methods Group and the Cochrane Non-Randomised Studies of Interventions Methods Group	
Lu et al. [26]		Intervention and observational studies				The Quality of Health Economic Studies (QHES) instrument: decision-analytic models		16		Extremely poor quality, poor quality, fair quality, and high quality				N/A				Generic	Validated			30 cost-effectiveness studies				Joshua and colleagues	
Nicholas et al. [32]		Observational studies				Modified version of NOS: cohort and case–control studies		6		Full-quality score, partial-quality score, and poor-quality score				Yes (*n* = 2)				Generic	Not available			N/A				Zeng and colleagues	
Omarini et al. [33]	Intervention and observational studies			STROBE: cohort, case–control, and cross-sectional studies			22					Suitable or poor			Yes (*n* = 4)	Generic					Not validated			N/A			N/A
Tahra et al. [34]	Observational studies			Assessing risk of bias in prevalence studies			10					low risk; moderate risk; high risk			Yes	Generic					Not validated			N/A			N/A
Fatoye et al. [35]	Observational studies			Assessing risk of bias in prevalence studies:			11					low risk; moderate risk; high risk (Modified version)			Yes	Generic					Not validated			N/A			Hoy and colleagues
Lin et al. [36]	Intervention and observational studies			The modified version of NOS: retrospective cohort studies and case series			7					low, moderate, or high quality			Yes (*n* = 2)	Generic					Not validated			N/A			Wells and colleagues
Alsadhan et al. [37]	Observational studies			The Joanna Briggs Institute Critical Appraisal tool for prevalence studies:			10					High, moderate, or low			N/A	Generic					Not validated			N/A			Joanna Briggs Institute
Erdos and Wild [38]	Intervention and observational studies			The Institute of Health Economics (IHE) Risk of Bias checklist			20					Low, moderate, or high			Yes (*n* = 8)	Generic					Not validated			N/A			IHE

*GRADE* The Grades of Recommendation, Assessment, Development, and Evaluation, *ROBINS-I* The Risk of Bias in Non-randomized Studies of Interventions, *N/A* Not Available, *STROBE* The Strengthening the Reporting of Observational Studies in Epidemiology Statement, *CASP* Critical Appraisal Skills Program

^**Ω**^These tools have not been independently published

### Non-summative four-point system

Non-summative four-point system is one of the included studies used a QA tool specific to real-world data [23]. The tool was developed by Wylde and colleagues, it is non-summative four-point system [19]. The tool consisted of four items used to assess selection bias (inclusion of consecutive patients and representativeness), bias due to missing data (follow-up rates) and bias due to inadequate consideration of confounding (multivariable or univariable analysis). Each item was rated as adequate, not adequate or not reported.

## Discussion

In this paper, we reviewed the methodological QA tools for SRs and MAs used in RWE studies. The included studies in our review were published between 2016 and 2021, this finding aligns with the period of recent surge of use of methodological QA tools in real-world data studies. However, there is inadequate use of QA tool in RWD compared to other SRs and MA using randomised clinical trial [39]. The use of appropriate QA tools in SRs and MAs involving RWD is needed to generate trustworthy conclusions and acceptable evidence and recommendations to be used in health care [40]. The key point that is considered in the process of utilising evidence from SRs and MAs is whether critical appraisal is carried out or not [41]. For example, the findings of a study [42] that assessed the methodological, reporting and evidence quality of SRs and MAs of total glucosides of paeony for rheumatoid arthritis indicated that although included studies summarised that glucoside of paeony was effective and safe in the treatment of rheumatoid arthritis, the methodological and reporting quality and the quality of evidence was poor. As a result, the study recommended that decision-makers should be prudent when using glucosides of paeony in treating rheumatoid arthritis. Hyrich [20] in highlighting the key role of RWD in rheumatology, noted that methodological challenges in analysing RWD is a significant challenge to generating reliable scientific output using RWD.

Variation was observed within the QA tools used in the SRs and MAs with regard to content of domains, checklist, and scales. For example, some of the QA criteria such as inclusion of consecutive patients, representativeness, and follow-up were frequently reported in QA tools. Thus, the absence of a specific QA tool can restrict the process of consistent and reliable appraisal for SRs and MAs studies that have used RWD. In the current review, the authors observed that some of the QA tools were adapted or modified [23, 32, 34, 36], whereas others used generic QA tools. Overall, little consensus was observed around the QA tools of the SRs and MAs for RWE studies.

The absence of a standard and specific QA tool for SRs and MAs involving RWE studies have resulted in the use of different types of QA tools that have been developed for other studies with a different methodology such as randomised controlled studies, cross-sectional studies. Except one [23], all the included studies for the current review have used different sets of QA tools that are generic. The tool developed by Evans and colleagues [23] was specific and consists of four items including inclusion of consecutive patients, representativeness, percentage of follow-up and minimisation of potential confounding. However, this QA tool was not validated, as its psychometric properties are lacking. Psychometric properties of a test are tests that identify and define critical aspects of an instrument that include its adequacy, relevance, and usefulness (or its validity) [43]. Other authors argued that there should be a QA tool which is specific to SRs and MAs for RWE that have been psychometrically tested for their feasibility, reliability, and validity [44].

The criteria to be used for QA in each type of tools are different and no specific tool covers all the methodological aspects. It is due to these methodological differences that relevant evaluation tools are developed based on the characteristics of different types of study. Some evaluation tools are, for example, used without recommendations for critical appraisal of evidence [45]. There are also many types of research methods such as before-after study (time series) and nested case–control study that do not have QA tools [46]. It is important that efforts should be made on developing QA tools for SRs and MAs of RWD.

This scoping review has certain strength and limitations. In this review, we used a systematic approach such as the screening of numerous data bases, and the involvement of multiple reviewers. Only studies conducted in English language were included, therefore, there is the possibility that some other relevant studies in other languages could have been excluded. Nevertheless, this review serves as a foundation for further work on QA tools in SRs and MA using RWD. Identification of appropriate QA tool for a specific type of study should be the priority for those utilising evidence from them. This is because it will be useful to increase the transparency and reproducibility of scientific work in real-world evidence. This study could be a foundation by way of summarising the QA tools while pointing out potential improvements to be adopted in the future.

## Conclusions

The findings of the present scoping review indicated that many different types of QA tools are currently used for RWD of SRs and MAs studies, while no validated and reliable specific tool currently exist. Thus, there is a need for a standardized and specific QA tool of SRs and MAs for RWD.

## Supplementary Information

Below is the link to the electronic supplementary material.

Supplementary file1 (DOCX 20 KB)

## Data Availability

All results from our analyses are published in the Supplementary Material, available at Rheumatology International online. Items/domains employed to the included studies and extracted by our investigators are available upon reasonable request.

## Declarations

### Disclaimer

No part of this review is copied or published elsewhere in whole or in part in any languages. The information in Appendix 1 are Items/domains employed to the included studies. They are specific criteria developed to be used for quality assessment.

### Data Sharing

All data related to this work are available in this research article.
