# Supplementary material for: A scoping review on quality assessment tools used in systematic reviews and meta-analysis of real-world studies
Source: Rheumatol Int. 2023 Jun 16;43(9):1573–81. doi: 10.1007/s00296-023-05354-x (PMC10348931; doi:10.1007/s00296-023-05354-x)
Supplement: Supplementary file 1 — Supplementary file1 (DOCX 20 KB) [file 296_2023_5354_MOESM1_ESM.docx]

**Appendix 1:** Items/domains employed to the included studies

| **Halling et al. [28]** | **Coratti et al. [25]** | **Kolmos et al. [30]** | **Evans et al. [31]** | **Alipour et al. [26]** |
| --- | --- | --- | --- | --- |
| 1.Was the research question or objective in this paper clearly stated?  2. Was the study population clearly specified and defined?  3. Was the participation rate of eligible persons at least 50%?  4.Were all the subjects selected or recruited from the same or similar populations (including the same time period)? Were inclusion and exclusion criteria for being in the study prespecified and applied uniformly to all participants?  5. Was a sample size justification, power description, or variance and effect estimates provided?  6. For the analyses in this paper, were the exposure(s) of interest measured prior to the outcome(s) being measured?  7.Was the timeframe sufficient so that one could reasonably expect to see an association between exposure and outcome if it existed?  8.For exposures that can vary in amount or level, did the study examine different levels of the exposure as related to the outcome (e.g., categories of exposure, or exposure measured as continuous variable)?  9.Were the exposure measures (independent variables) clearly defined, valid, reliable, and implemented consistently across all study participants?  10.Was the exposure(s) assessed more than once over time?  11.Were the outcome measures (dependent variables) clearly defined, valid, reliable, and implemented consistently across all study participants?  12.Were the outcome assessors blinded to the exposure status of participants?  13.Was loss to follow-up after baseline 20% or less?  14.Were key potential confounding variables measured and adjusted statistically for their impact on the relationship between exposure(s) and outcome(s)? | 1.Selection bias caused by the inadequate selection of participants 2. Selection bias caused by the inadequate confirmation and consideration of confounding variable 3. Performance bias caused by the inadequate measurement of exposure 4. Detection bias caused by the inadequate blinding of outcome assessments 5. Attrition bias caused by the inadequate handling of incomplete outcome data 6. Reporting bias caused by the selective reporting of outcomes | 1. Did the study address a clearly focused issue? 2. Was the cohort recruited in an acceptable way? 3. Was the exposure accurately measured to minimise bias? 4. Was the outcome accurately measured to minimise bias? 5. (a) Have the authors identified all important confounding factors? 5. (b) Have they taken account of the confounding factors in the design and/or analysis? 6 (a)Was the follow up of subjects complete enough? 6. (b)Was the follow up of subjects long enough? 7. What are the results of this study? 8. How precise are the results? 9. Do you believe the results? 10.Can the results be applied to the local population? 11.Do the results of this study fit with other available evidence? 12.What are the implications of this study for practice? | 1) Inclusion of consecutive patients 2) Representativeness (multicentre adequate) 3) Percentage follow-up (> 80% adequate) 4) Minimisation of potential confounding (multivariable analysis adequate) | **Selection** 1) Representativeness of the exposed cohort a) truly representative of the average __ (describe) in the community Ø b) somewhat representative of the average __ in the community Ø c) selected group of users eg nurses, volunteers d) no description of the derivation of the cohort 2) Selection of the non-exposed cohort a) drawn from the same community as the exposed cohort Ø b) drawn from a different source c) no description of the derivation of the non-exposed cohort 3) Ascertainment of exposure a) secure record (eg surgical records) Ø; b) structured interview Ø; c) written self-report; d) no description; 4) Demonstration that outcome of interest was not present at start of study; a) yes Ø; b) no Comparability **Comparability of cohorts on the basis of the design or analysis** a) study controls for__ (select the most important factor) Ø b) study controls for any additional factor Ø (This criteria could be modified to indicate specific control for a second important factor.)  **Outcome** 1) Assessment of outcome; a) independent blind assessment Ø; b) record linkage Ø; c) self-report. d) no description 2) Was follow-up long enough for outcomes to occur a) yes (select an adequate follow up period for outcome of interest) Ø b) no 3) Adequacy of follow up of cohorts a) complete follow up - all subjects accounted for Ø b) subjects lost to follow up unlikely to introduce bias - small number lost - > _ % (select an  adequate %) follow up, or description provided of those lost) Ø c) follow up rate < __% (select an adequate %) and no description of those lost d) no statement |

**Appendix 1:** Continued

| **van der List et al. [31]** | **Lin et al. [37]** | **Nicholas et al. [33]** | **Lu et al. [27]** |
| --- | --- | --- | --- |
| 1. A stated aim of the study  2. Inclusion of consecutive patients 3. Prospective collection of data  4. Endpoint appropriate to the study aim 5. Unbiased evaluation of endpoints  6. Follow-up period appropriate to the major endpoint  7. Loss to follow up not exceeding 5% And in the case of comparative studies 8. A control group having the gold standard intervention  9. Contemporary groups  10. Baseline equivalence of groups  11. Prospective calculation of the sample size  12. Statistical analyses adapted to the study design | 1.Representativeness of exposed cohort 2. Selection of nonexposed cohort 3. Ascertainment of exposure 4. Outcome not present at start of study 5. Assessment of outcome 6. Length of follow-up 7. Adequacy of follow-up | Study design and patient selection Ascertainment of intervention/validity of study design; Patient selection; Outcome not present at start Outcome evaluation Appropriate measure of adherence/persistence; Adequate/ appropriate duration of follow-up; All patients accounted for followed up | 1.Was the study objective presented in a clear, specific, and measurable manner?  2.Were the perspective of the analysis (societal, third-party payer, etc.) and reasons for its selection stated?  3.Were variable estimates used in the analysis from the best available source (i.e., randomized control trial - best, expert opinion - worst)?  4. If estimates came from a subgroup analysis, were the groups prespecified at the beginning of the study?  5.Was uncertainty handled by (1) statistical analysis to address random events, (2) sensitivity analysis to cover a range of assumptions?  6.Was incremental analysis performed between alternatives for resources and costs?  7.Was the methodology for data abstraction (including the value of health states and other benefits) stated?  8.Did the analytic horizon allow time for all relevant and important outcomes? Were benefits and costs that went beyond 1 year discounted (3% to 5%) and justification given for the discount rate?  9.Was the measurement of costs appropriate and the methodology for the estimation of quantities and unit costs clearly described?  10.Were the primary outcome measure(s) for the economic evaluation clearly stated and did they include the major short-term was justification given for the measures/scales used? 11.Were the health outcomes measures/scales valid and reliable? If previously tested valid and reliable measures were not available, was justification given for the measures/scales used?  12.Were the economic model (including structure), study methods and analysis, and the components of the numerator and denominator displayed in a clear, transparent manner?  13. Were the choice of economic model, main assumptions, and limitations of the study stated and justified?  14. Did the author(s) explicitly discuss direction and magnitude of potential biases?  15. Were the conclusions/recommendations of the study justified and based on the study results?  16. Was there a statement disclosing the source of funding for the study? |

**Appendix 1:** Continued

| **Tahra et al. [35]** | **Fatoye et al. [36]** | **Lin et al. [37]** | **Alsadhan et al. [38]** | **Rahhal et al. [32]** |
| --- | --- | --- | --- | --- |
| 1. Was the study’s target population a close representation of the national population in relation to relevant variables, e.g. age, sex, occupation?; 2. Was the sampling frame a true or close representation of the target population?; 3,Was some form of random selection used to select the sample, OR, was a census undertaken? 4. Was the likelihood of non-response bias minimal? 5. Were data collected directly from the subjects (as opposed to a proxy)? 6. Was an acceptable case definition used in the study? 7.Was the study instrument that measured the parameter of interest (e.g. prevalence of low back pain) shown to have reliability and validity (if necessary)? 8. Was the same mode of data collection used for all subjects? 9. Were the numerator(s) and denominator(s) for the parameter of interest appropriate; 10. Summary on the overall risk of study bias | 1. Was the study’s target population a close representation of the national population in relation to relevant variables, e.g. age, sex, occupation? 2. Was the sampling frame a true or close representation of the target population? 3,Was some form of random selection used to select the sample, OR, was a census undertaken? 4. Was the likelihood of non-response bias minimal? 5. Were data collected directly from the subjects (as opposed to a proxy)? 6. Was an acceptable case definition used in the study? 7. Was the study instrument that measured the parameter of interest (e.g. prevalence of low back pain) shown to have reliability and validity (if necessary)? 8. Was the same mode of data collection used for all subjects? 9. Was the length of the shortest prevalence period for the parameter of interest appropriate? 10. Were the numerator(s) and denominato r(s) for the parameter of interest appropriate 11. Summary on the overall risk of study bias | **Selection**: 1. Representativeness of the exposed cohort 2. Ascertainment of exposure 3. Outcome not present at start of study **Outcome:**  4.Assessment of outcome 5. Length of follow-up 6. Adequacy of follow-up **Total quality score** | 1.Was the sample frame appropriate to address the target population? 2.Were study participants sampled in an appropriate way? 3.Was the sample size adequate? 4.Were the study subjects and the setting described in detail? 5.Was the data analysis conducted with sufficient coverage of the identified sample? 6.Were valid methods used for the identification of the condition? 7.Was the condition measured in a standard, reliable way for all participants? 8.Was there appropriate statistical analysis? 9.Was the response rate adequate, and if not, was the low response rate managed appropriately? 10.**Overall appraisal**: Include □ Exclude □ Seek further info □ | 1. Bias due to confounding  2. Bias in selection of participants into the study 3. Bias in classification of interventions 4. Bias due to deviations from intended interventions 5. Bias due to missing data 6. Bias in measurement of outcomes 7. Bias in selection of the reported result |

**Appendix 1:** Continued

| **Omarini et al. [34]** | **Erdos & Wild. [39]** |
| --- | --- |
| 1.(a) Indicate the study’s design with a commonly used term in the title or the abstract  (b) Provide in the abstract an informative and balanced summary of what was done and what was found  2.Explain the scientific background and rationale for the investigation being reported  3.State specific objectives, including any prespecified hypotheses  4.Present key elements of study design early in the paper  5.Describe the setting, locations, and relevant dates, including periods of recruitment, exposure, follow-up, and data collection  6.(a) Give the eligibility criteria, and the sources and methods of selection of participants. Describe methods of follow-up  (b) For matched studies, give matching criteria and number of exposed and unexposed  7.Clearly define all outcomes, exposures, predictors, potential confounders, and effect modifiers. Give diagnostic criteria, if applicable  8. For each variable of interest, give sources of data and details of methods of assessment (measurement). Describe comparability of assessment methods if there is more than one group  9.Describe any efforts to address potential sources of bias  10.Explain how the study size was determined  11.Explain how quantitative variables were handled in the analyses. If applicable, describe which groupings were chosen and why  12.(a) Describe all statistical methods, including those used to control for confounding  (b) Describe any methods used to examine subgroups and interactions; (c) Explain how missing data were addressed  (d) If applicable, explain how loss to follow-up was addressed; (e) Describe any sensitivity analyses  13(a) Report numbers of individuals at each stage of study; (b) Give reasons for non-participation at each stage  (c) Consider use of a flow diagram  14(a) Give characteristics of study participants (e.g. demographic, clinical, social) and information on exposures and potential confounders; (b) Indicate number of participants with missing data for each variable of interest  (c) Summarise follow-up time (e.g., average and total amount)  15.Report numbers of outcome events or summary measures over time  16(a) Give unadjusted estimates and, if applicable, confounder-adjusted estimates and their precision (e.g. 95% confidence interval). Make clear which confounders were adjusted for and why they were included  (b) Report category boundaries when continuous variables were categorized  (c) If relevant, consider translating estimates of relative risk into absolute risk for a meaningful time period  17.Report other analyses done—e.g. analyses of subgroups and interactions, and sensitivity analyses  18.Summarise key results with reference to study objectives  19.Discuss limitations of the study, taking into account sources of potential bias or imprecision. Discuss both direction and magnitude of any potential bias  20.Give a cautious overall interpretation of results considering objectives, limitations, multiplicity of analyses, results from similar studies, and other relevant evidence  21.Discuss the generalisability (external validity) of the study results  22.Give the source of funding and the role of the funders for the present study and, if applicable, for the original study on which the present article is based | **Study objective**  1.Was the hypothesis/aim/objective of the study clearly stated?  Study design  2. Was the study conducted prospectively?  3.Were the cases collected in more than one centre?  4.Were patients recruited consecutively?  **Study population**  5. Were the characteristics of the patients included in the study described?  6. Were the eligibility criteria (i.e. inclusion and exclusion criteria) for entry into the study clearly stated?  7. Did patients enter the study at a similar point in the disease?  Intervention and co-intervention  8.Was the intervention of interest clearly described?  9. Were additional interventions (co-interventions) clearly described?  **Outcome measure**  10. Were relevant outcome measures established a priori?  11. Were outcome assessors blinded to the intervention that patients received?  12. Were the relevant outcomes measured using appropriate objective/subjective methods?  13. Were the relevant outcome measures made before and after the intervention?  **Statistical analysis**  14. Were the statistical tests used to assess the relevant outcomes appropriate?  **Results and conclusions**  15. Was follow-up long enough for important events and outcomes to occur?  16. Were losses to follow-up reported?  17. Did the study provided estimates of random variability in the data analysis of relevant outcomes?  18. Were the adverse events reported?  19. Were the conclusions of the study supported by results?  Competing interests and sources of support  20. Were both competing interests and sources of support for the study reported? |

**Appendix 2:** Search strategy

S9 S7 AND S8

S8 S5 OR S6

S7 S1 OR S2 OR S3 OR S4

S6 TI meta-analysis

S5 TI Systematic review

S4 TI Registries

S3 TI Electronic health records

S2 TI Routinely collected data

S1 TI Real-world data
